# Some Remarks on Gunshot Wounds

**Published:** 1895-04

**Authors:** H. McHatton

**Affiliations:** Macon, Ga.


### SOME REMARKS ON GUNSHOT WOUNDS.

By H. McHATTON, M.D.,

MACON, GA.

CASE I.—Wound about two inches in diameter opposite junction of middle and upper third of femur, cutting through sartorius and part of rectus femoris; inner aspect of wound just external to femoral artery. Machine-loaded shell, one and a quarter ounces number five shot, three and a half drachms of powder, penetration sufficient to drive some shot as far as skin of posterior aspect of thigh. Temporary loss of extension power of leg, which was fully recovered in about six months.

CASE II.—Distribution of shot on external aspect of arm, extending from junction of middle and lower third of radius, few shot passing through arm, and some eighteen or twenty penetrating chest wall. Shell same as in Case I. Slight hemoptysis. Perfect recovery.

CASE III.—Distribution of shot all over posterior inner aspect of upper third of thigh. Shell supposed to be same as above. Constitutional symptoms of moderate severity, some impairment of sciatic nerve, unpleasant neuralgic symptoms. Complete recovery in four months.

CASE IV.—Wound, as far as could be judged, over exit of femoral, just below Poupart's ligament, .38 caliber pistol ball. Large hematoma present; had been considerable hemorrhage; next day limb was enormously swollen, which swelling reached its maximum about the third day; constitutional symptoms of unusual severity; considerable suppuration from original wound. Complete recovery.

CASE V.—Wound just over middle third of femoral artery, .22 caliber rifle ball. No constitutional symptoms. Complete recovery.

CASE VI.—Wound just to right of mental protuberance of lower jaw, splitting up body of bone as far as angle; .32 caliber pistol ball. No constitutional symptoms. Rapid and complete recovery.

These cases are from six months to four years old, and in none have there been any secondary symptoms caused by the position of the missile. They cover about all sizes of projectiles that we are liable to find in private practice. The shotgun wounds are of especial interest, as each of these patients is at present carrying, according to the Tatham standard, two hundred shot, making six hundred in the three cases. One of these cases is four years old, another three, and the other six months. The only discomfort experienced by any of them is Case I, who says that when sitting down, if he slips backward or forward in the chair, he has some discomfort from the subcutaneous shot on the posterior aspect of the thigh, which can of course be easily remedied. In Case II I expected a stiff elbow, but got complete motion.

The treatment pursued in these cases was simply recognizing the wounds as probably aseptic, treating them as such and fixing as near as possible the parts. The point to which I wish to draw especial attention is that in no case was there any effort made to locate the position of the projectile, and the wound was not interfered with farther than to cleanse the surrounding tissues and put on an antiseptic dressing.

I do not go fully into the clinical history of these cases, as it would have but little bearing on the point which I wish to make, but will state that the convalescence in each case was as rapid and uneventful as could be expected in a wound of the given type.

For the past few years there has been an increasing tendency to let gunshot wounds severely alone, and the results have been good. The latest bacteriological investigations on this line have added their weight to this position, by showing that modern bullets are practically aseptic. Of course we are liable to have a wound infected by other bodies being carried into it, such as clothing, but from year to year, with the increasing initial velocity and decreasing caliber of modern fire-arms, this factor is growing constantly less. In almost every instance of gunshot wound the passage of the projectile is what has done the damage, and its position is a matter of no importance. In the few cases where it is a factor, its position can, as a rule, be made out as distinctly by the signs and symptoms of the case as by the introduction of some foreign body into the wound, and certainly with much more safety to the patient. Popular, and, to an unfortunately large extent, professional, ideas in these cases seem to hang on the location of the ball, and in my estimation many a wound of trivial importance has eventually caused death from the effectual or ineffectual efforts to locate and remove the ball. I can recall scenes around the bedside of these patients that, to say the least, were not pleasant—probes and catheters from pocket-cases and fingers from all sorts of cases, with usually no effort at asepsis, but sometimes an apology in that line, poked and prodded around in a wound (usually without the use of an anesthetic, thereby vastly increasing any existing shock), when there was not a sign or symptom of any description demanding interefrence, excepting the self-evident fact that the patient had been shot. Among all these cases I do not recall a single one where any benefit was or could have been derived. In one case, in fact, after a most prolonged effort at locating the ball, the wound of exit was subsequently found. Excepting in a very small percentage of cases, where the immediate removal of the ball is imperative, should its position be an element of danger or discomfort, its removal at other times and under other surroundings will be found much safer to the patient. I think it is a safe rule for us, in all cases of gunshot wounds, to fix the parts, dress the wound antiseptically, and let it alone, unless some sign or symptom absolutely demands interference, which signs or symptoms would of course be evident in cases of injuries to blood-vessels, viscera, or other vital organs. The element of fixation in these cases is one of great importance. Not long since I saw a report from a western hospital of over twenty consecutive recoveries from wounds of the thoracic cavity, several of these cases being of multiple wounds. They were treated with an antiseptic dressing under a plaster of Paris jacket.

Another question of importance is that the majority of these cases eventually involve medico-legal questions, as it is pleasant to know when we may land in the hands of some lawyer who makes a specialty of this line of work. I will close by quoting the following from Witthaus & Becker's Medico-Legal Jurisprudence, which may be considered the coming authority in this country:

"The average bullet wound, however, is sterile as far as infection from the wound is concerned, and in accordance with this view of its usual innocence there need be no longer the clamor for removal of the missile which the fears of previous generations have nearly always called for, and the practice among military surgeons of to-day is rather to let the bullet remain where it lodged than to make a more serious wound for its removal. Exceptions to this rule occur only where operation is called for on account of injury done by the bullet while still in motion. It is also held to be a violation of simple physiological and surgical rules to probe or carelessly search for a bullet whose location cannot be made out from the study of signs and symptoms in a given case. The act of probing breaks up blood-clot, often brings on fresh hemorrhage, is in a majority of cases unsatisfactory, frequently introduces septic elements from without, and really gives little, if any, more information than can be gathered from a study of the case without the use of the probe. If every ordinary bullet wound which did not call for immediate operation because of injury to some essential or vital part, such as a large blood-vessel or nerve tissue, or some of the viscera, were antiseptically and hermetically sealed at the very outset, there would be a much smaller percentage of death from gunshot wounds, either in civil or military practice, than now obtains, and it might be a matter upon which to go to the jury whether the violation of such rules, to-day, does not mitigate the offense of the accused."

